# Rare 15q21.1q22.31 Duplication Due to a Familial Chromosomal Insertion and Diagnostic Investigation in a Carrier of Balanced Chromosomal Rearrangement and Intellectual Disability

**DOI:** 10.3390/genes14040885

**Published:** 2023-04-09

**Authors:** Carolina Gama Nascimento, Joana Rosa Marques Prota, Ilária Cristina Sgardioli, Samira Spineli-Silva, Nilma Lúcia Viguetti Campos, Vera Lúcia Gil-da-Silva-Lopes, Társis Paiva Vieira

**Affiliations:** Department of Translational Medicine—Medical Genetics and Genomic Medicine, School of Medical Sciences, State University of Campinas, Campinas 13083-887, SP, Brazil

**Keywords:** 15q21.1q22.31 duplication, familial chromosomal insertion, balanced chromosomal rearrangement, *CYP19A1* gene duplication

## Abstract

Insertions are rare balanced chromosomal rearrangements with an increased risk of imbalances for the offspring. Moreover, balanced rearrangements in individuals with abnormal phenotypes may be associated to the phenotype by different mechanisms. This study describes a three-generation family with a rare chromosomal insertion. G-banded karyotype, chromosomal microarray analysis (CMA), whole-exome sequencing (WES), and low-pass whole-genome sequencing (WGS) were performed. Six individuals had the balanced insertion [ins(9;15)(q33;q21.1q22.31)] and three individuals had the derivative chromosome 9 [der(9)ins(9;15)(q33;q21.1q22.31)]. The three subjects with unbalanced rearrangement showed similar clinical features, including intellectual disability, short stature, and facial dysmorphisms. CMA of these individuals revealed a duplication of 19.3 Mb at 15q21.1q22.31. A subject with balanced rearrangement presented with microcephaly, severe intellectual disability, absent speech, motor stereotypy, and ataxia. CMA of this patient did not reveal pathogenic copy number variations and low-pass WGS showed a disruption of the *RABGAP1* gene at the 9q33 breakpoint. This gene has been recently associated with a recessive disorder, which is not compatible with the mode of inheritance in this patient. WES revealed an 88 bp deletion in the *MECP2* gene, consistent with Rett syndrome. This study describes the clinical features associated with the rare 15q21.1–q22.31 duplication and reinforces that searching for other genetic causes is warranted for individuals with inherited balanced chromosomal rearrangements and abnormal phenotypes.

## 1. Introduction

Insertions are rare balanced chromosomal rearrangements. Large chromosomal insertions, detected by karyotype analysis, have an estimated incidence of approximately 1:80,000 to 1:10,000 live births [1]. However, more recent studies using microarray analysis in conjunction with fluorescence in situ hybridization (FISH) suggest that the incidence is likely to be higher, in 1:3380 to 1:5200 of live births [2]. Most of the carriers of balanced insertions have a normal phenotype. However, these individuals have an increased risk of transmitting the insertion in an unbalanced form, resulting in segmental monosomies or trisomies [1,3]. These imbalances are usually associated with abnormal phenotypes, including multiple congenital anomalies, intellectual disability, and dysmorphic features, depending on the size and gene content of the segment involved [4].

Individuals with duplications from 15q21.1 to 15q22.31 have been described in a few articles to date [5,6,7,8,9]. In addition, seven individuals with duplications ranging from 8.15 Mb to 22.26 Mb, encompassing 15q15.1 to 15q24.1 genomic regions, were included in the DatabasE of genomiC variation and Phenotype in Humans using Ensembl Resources—DECIPHER [10]. They showed varied phenotypes, with recurrence of short stature, delayed speech and language development, and low-set ears. To date, duplications with breakpoints exactly at 15q21.1 and 15q22.31 have not been described.

Although most individuals with balanced rearrangements, including insertions, show normal phenotype [11], some individuals with balanced chromosomal rearrangements can have abnormal phenotype due to different mechanisms [12], including gene disruption at the breakpoints or positional effect, which can cause loss of function of dose-sensitive genes, and submicroscopic imbalances close to the breakpoints [2]. These genomic imbalances are found in up to 30–50% of individuals with balanced chromosomal rearrangements and abnormal phenotype when investigated by chromosomal microarray analysis (CMA) [11]. In this study, we describe a rare familial interchromosomal insertion, the phenotype of individuals with duplication at 15q21.1–15q22.31 who inherited the unbalanced form of the insertion, and the complementary diagnostic investigation of a carrier of the balanced rearrangement with intellectual disability.

## 2. Materials and Methods

### 2.1. Cytogenetic Analysis

G-banded chromosome analyses were performed on cultured lymphocytes from peripheral blood, with a resolution of approximately 400–550 bands, according to standard protocols.

### 2.2. Chromosomal Microarray Analysis (CMA)

Genomic DNA was extracted from peripheral blood using standard protocols. CMA was performed using the SNP array 6.0 from Affymetrix^®^ (Thermo Fisher Scientific Inc.—Life Technologies, Carlsbad, CA, USA), according to the manufacturer’s instructions. Results were analyzed using the Genotyping Console v. 3.0.2 (HMM—Affymetrix^®^) and the Chromosome Analysis Suite (ChAS version 3.3.0.139 (r10838)—Affymetrix^®^) (Thermo Fisher Scientific Inc.—Life Technologies, Carlsbad, CA, USA) with the hg19 human genome build. Losses and gains were considered for analysis with a minimum number of 25 probes as a reliable indicator for loss and 50 probes for gain, as well as 500 probes and a size of 1500 kbp as a reliable indicator of homozygous regions.

### 2.3. Low-Pass Whole Genome Sequencing (WGS) and Whole Exome Sequencing (WES) 

Low-pass WGS and WES were performed for subject IV-12 to map the rearrangement breakpoints and to investigate other possible genetic causes, respectively. Low-pass WGS and breakpoint mapping were performed as previously described [13].

WES was performed using the Agilent SureSelect Target Enrichment V5 capture kit (Agilent Technologies, Santa Clara, CA, USA) and the Illumina HiSeq platform (Illumina, San Diego, CA, USA), as previously described [14]. Data analysis, including annotation and variant classification, was performed at the Genomic Diagnostics Division from the Department of Human Genetics of the Radboud University Medical Center, Nijmegen, Netherlands, according to the recommendations of the American College of Medical Genetics and Genomics [15].

## 3. Results

**Figure 1:**
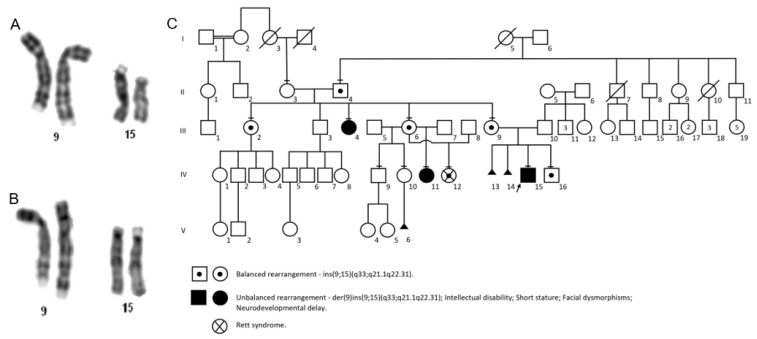
(**A**) Partial G-banded karyotype of chromosomes 9 and 15, balanced insertion—ins(9;15)(q33;q21.1q22.31). (**B**) Partial G-banded karyotype of chromosomes 9 and 15, unbalanced insertion—der(9)ins(9;15)(q33;q21.1q22.31)—resulting in duplication of the q21.1q22.31 region of chromosome 15. (**C**) Five-generation pedigree of the family showing the affected individuals and the transmission of the chromosomal rearrangement in three generations.

G-banded chromosome analyses revealed a rare chromosomal insertion, ins(9;15)(q33;q21.1q22.31), segregating in three generations of the family (Figure 1C). Subjects II-4, III-2, III-6, III-9, IV-12 and IV-16 had the balanced rearrangement—ins(9;15)(q33;q21.1q22.31) (Figure 1A), and subjects III-4, IV-11 and IV-15 had the unbalanced rearrangement—der(9)ins(9;15)(q33;q21.1q22.31) (Figure 1B).

### 3.1. Cases Presentation

#### 3.1.1. Subject 1 (III-4)

**Figure 2:**
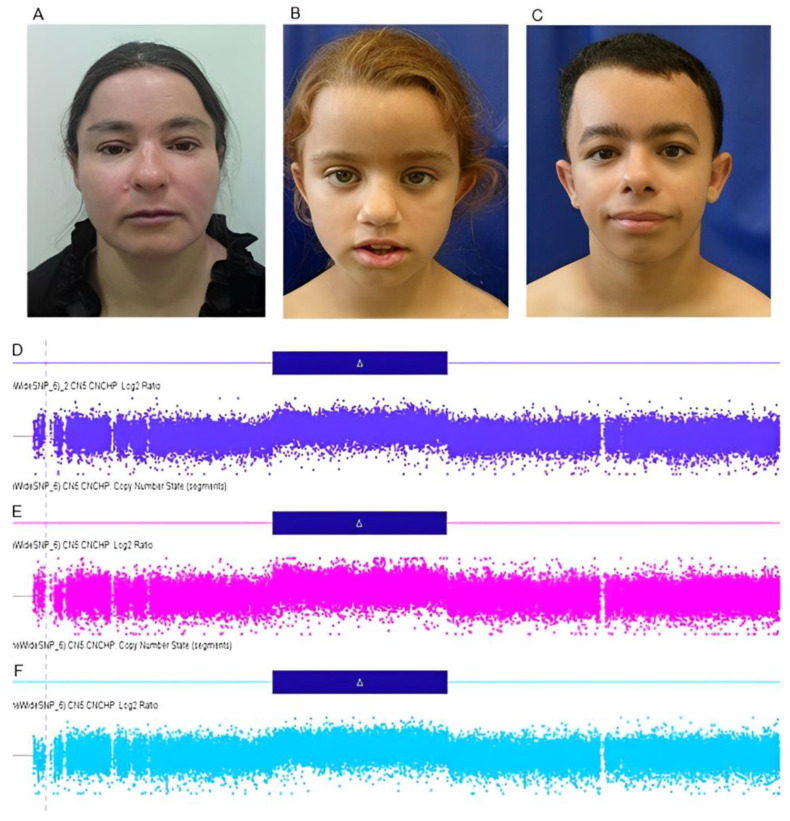
(**A**–**C**) Affected individuals harboring the unbalanced rearrangement, with a 19.3 Mb duplication of the q21.1q22.31 region of chromosome 15, with facial dysmorphisms: discrete synophrys, upslanted palpebral fissure, low-set ears, bifid nasal tip, downturned corners of mouth, and retrognathia. Subject 1 (III-4) (**A**), subject 2 (IV-11) (**B**), and subject 4 (IV-15) (**C**). (**D**–**F**) Log2ratio graphics, obtained by CMA, of part of the long arm of chromosome 15, showing a 19.3 Mb duplication at 15q21.1q22.31 (46487891_65795296—GRCh37) in the individuals with unbalanced insertion.

A female patient was referred for genetic evaluation at 38 years of age for presenting with a family history of chromosomal rearrangement, in addition to intellectual disability, short stature, and facial dysmorphisms. She was the third child of healthy and non-consanguineous parents. She was born by natural home birth, weighing 3.0 kg (−0.52 SD), and had no weight gain during the neonatal period. She walked unsupported at 3 years, spoke complete sentences at 7 years, and was diagnosed with neurodevelopmental, speech, and language delay, evolving with intellectual disability. At 38 years of age, her weight was 41.5 kg (−2.32 SD), her length was 130.6 cm (−4.98 SD), and her head circumference was 50.5 cm (−2.83 SD). The dysmorphological evaluation showed short stature, flat occiput, synophrys, upslanted palpebral fissure, bifid nasal tip, downturned corners of mouth, retrognathia, rhizomelic limb shortening, and bilateral cubitus valgus (Figure 2A). Moreover, she had congenital heart disease and premature ovarian insufficiency at around 37 years of age. Karyotype and CMA analyses showed an unbalanced chromosomal rearrangement—46,XX,der(9)ins(9;15)(q33;q21.1q22.31)[20] (Figure 1B)—and a duplication of 19.3 Mb at 15q21.1q22.31 (46,487,891_65,790,216—GRCh37) (Figure 2D), respectively. Her father’s karyotype was 46,XY,ins(9;15)(q33;q21.1q22.31)[20].

#### 3.1.2. Subject 2 (IV-11)

A female patient was referred for genetic evaluation at 9 years of age for presenting with neurodevelopmental and growth delay, short stature, and facial dysmorphisms. She was the third child on the mother’s side, and the only daughter of a young, healthy, non-consanguineous couple. The mother had two other healthy previous children, and a younger daughter (Subject IV-12) also with neurodevelopmental delay (Figure 1C). The prenatal period was uneventful. The patient was born by cesarean section with the following measurements: weight 2.8 kg (−0.99 SD), length 47 cm (−1.15 SD), and head circumference 34 cm (+0.10 SD). The patient walked unsupported at 3 years, spoke her first words at 4 years, and was diagnosed with neurodevelopmental, speech, and language delay, evolving into intellectual disability. Anthropometrical data at 9 years of age revealed height of 116.8 cm (−2.93 SD), weight of 20.2 kg (−2.55 SD), and head circumference of 48 cm (−3.3 SD). The dysmorphological evaluation showed V-shaped hairline on the forehead and trident-shaped on the nape, flat occiput, discrete synophrys, low-set ears, prominent ear, flat nasal root, bifid nasal tip and anteverted nostrils, thin upper lip vermilion, malar flattening, and short thumb (Figure 2B). She also had recurrent urinary tract infections. Complementary investigations included normal echocardiogram, abdominal ultrasound, and bone age at 8 years old compatible with 7 years and 9 months. The ophthalmological evaluation showed amblyopia. Menarche occurred at 12 years old with persistent irregularity until the last consultation, at 19 years of age; it was suggestive of secondary amenorrhea. Gynecological and endocrinological investigations were normal. At this age, she was still illiterate and dependent in activities of daily living. Karyotype and CMA analyses showed an unbalanced chromosomal rearrangement—46,XX,der(9)ins(9;15)(q33;q21.1q22.31)[20] (Figure 1B)—and a duplication of 19.3 Mb at 15q21.1q22.31 (46,487,891–65,790,216—GRCh37) (Figure 2E), respectively, similar to her aunt (Subject 1—III-4). Her mother’s karyotype was 46,XX,ins(9;15)(q33;q21.1q22.31)[20] (Figure 1A).

#### 3.1.3. Subject 3 (IV-12) 

**Figure 3:**
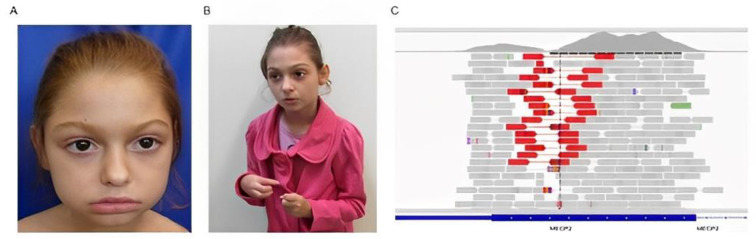
(**A**,**B**) Subject 3 (IV-12), harboring an inherited balanced chromosomal insertion, with microcephaly, highly arched eyebrows, bulbous nose, thin upper lip vermilion, and everted lower lip vermilion; (**C**) Image taken from the Integrative Genome Viewer (IGV), showing a region with red reads in the exon 3 of the *MECP2* gene, which corresponds to an 88 bp deletion in this gene (chrX:153296091-153296179—GRCh37). Variant allele frequency (VAF) = 0.40; depth of coverage (SD) = 111.

A female patient was referred for genetic evaluation at 4 years of age for presenting with intellectual disability, absent speech, and motor stereotypy. She was the fourth child on the mother’s side, and the only daughter of a young, healthy, non-consanguineous couple. She had an older sister with intellectual disability, but with a distinct phenotype (Subject 2—IV-11). The patient was born by cesarean delivery with the following measurements: weight 3.0 kg (−0.52 SD), and length 49 cm (−0.08 SD). In the first month of life, she had two episodes of cyanosis. She had a stiff neck at 3 months and sat unsupported at 5 months. At 9 months she started speaking words and walked unsupported at 2 years of age. At 2 years, she suffered an accident and lost the ability to speak. The patient was diagnosed with neurodevelopmental delay and intellectual disability. At 4 years of age, she developed atonic seizures. At this age, her anthropometrical data were height 102.1 cm (−0.97 SD), weight 13.4 kg (−1.84 SD), and head circumference 45 cm (−3.29 SD). The clinical and dysmorphological evaluation showed absent speech, highly arched eyebrows, bulbous nose, thin upper lip vermilion, everted lower lip vermilion, mid-line motor stereotypy, upper limbs and pedal edema, pes planus, gait ataxia, aggressive behavior, and hyperactivity (Figure 3A,B). The ophthalmological evaluation was normal. Electroencephalogram showed diffuse epileptiform activity. At 18 years old, her anthropometrical data were height 147.5 cm (−2.36 SD), weight 31.4 kg (−4.37 SD), and head circumference 49.2 cm (−3.72 SD). She walked some steps without support, spoke four words, showed little gestural communication, and had bladder–bowel incontinence. Seizures were controlled, and she was under investigation for cardiac arrhythmia. Karyotype analysis identified the balanced chromosomal insertion, similar to that of her mother and other family members—46,XX,ins(9;15)(q33;q21.1q22.31)[20] (Figure 1A). CMA of the patient and her mother did not reveal pathogenic genomic imbalances.

Low-pass WGS, performed to map the insertion breakpoints, identified the breakpoint on chromosome 9 at the genomic position 125,833,639 (GRCh37) within 9q33 and the breakpoints on chromosome 15 at the genomic positions 46,485,297 (GRCh37) within 15q21.1, and 65,811,644 (GRCh37) within 15q22.31. The breakpoint at 9q33 disrupted the *RABGAP1* gene. There were no genes on the breakpoints of chromosome 15. Further, WES revealed an 88 bp deletion in the *MECP2* gene—MECP2:NM_001110792.2:exon3:c.1136_1224del:p.(His379Argfs*8), located in Xq28 (Figure 3C). This variant is pathogenic and associated with Rett syndrome. Clinical criteria for Rett Syndrome (DSM—IV) applied for this subject revealed an atypical form, since, at her last evaluation, she showed partial loss of acquired purposeful hand skills, gait abnormalities, and partial loss of acquired spoken language; however, she was still able to walk unsupported and did not show stereotypic hand movements.

#### 3.1.4. Subject 4 (IV-15)

A male patient was referred for genetic evaluation at 9 years of age for presenting with neurodevelopmental and growth delay, congenital heart disease, facial dysmorphisms, and velopharyngeal insufficiency. He was the first child of healthy, young, and non-consanguineous parents. The mother reported two miscarriages before the proband’s pregnancy, and there was recurrence of these clinical features in other family members. During the pregnancy, his mother attended prenatal care, and there were no complications. The patient was born by natural delivery at 35 weeks with the following measurements: weight 1.59 kg (−2.14 SD), length 41 cm (−2.42 SD), and head circumference 29 cm (−3 SD). At two months of age, congenital mitral stenosis and bilateral inguinal hernia were identified. He also developed generalized tonic–clonic seizures. He spoke his first words at 9 months and walked unsupported at 13 months, but evolved with neurodevelopmental, speech and language delay, and intellectual disability. Anthropometrical data at 9 years of age were weight 17.6 kg (−3.59 SD), height 105 cm (−4.68 SD), and head circumference 49.5 cm (−2.52 SD). The dysmorphological evaluation showed synophrys, convergent strabismus, epicanthus, low-set ears, wide nasal base, prominent columella, prominent nasal tip and anteverted nares, bifid nasal tip, smooth philtrum, high palate, wide mouth, dental malocclusion, velopharyngeal insufficiency, retrognathia, short and wide neck, thickened skin, diastasis recti, skeletal muscle hypertrophy, rhizomelic limb shortening, mild camptodactyly of finger, shortening of 4th and 5th metacarpal, and cubitus valgus (Figure 2C). X-rays showed Wormian bones, mild hypoplasia of iliac bones, and L1 accessory rib at the right. Magnetic resonance of the brain was normal. He also had recurrent urinary tract infections (urinary ultrasound was normal), and hypothyroidism. Karyotype analysis showed an unbalanced chromosomal rearrangement—46,XY,der(9)ins(9;15)(q33;q21.1q22.31)[20] (Figure 1B). Karyotype analysis of the mother showed a balanced insertion—46,XX,ins(9;15)(q33;q21.1q22.31)[20] (Figure 1A). The patient’s CMA revealed a 19.3 Mb duplication at 15q21.1q22.31 (46,487,891–65,790,216—GRCh37) (Figure 2F). At 12 years of age, he had growth delay, short stature, muscular hypertrophy, and accelerated skeletal maturation identified by bone age X-ray, showing bone age consistent with 14.8 years by the TW2 method (20 bones) and 16 years by the Greulich–Pyle method. The endocrinological investigation was normal, besides hypothyroidism. At 21 years of age, anthropometrical data were height 131.5 cm (−6.17 SD), weight 36 kg (−4.79 SD), and head circumference 52.5 cm (−2.76 SD). He was independent in daily life activities but was still semi-illiterate.

## 4. Discussion

Large chromosomal insertions are very rare chromosomal rearrangements, and few familial cases have been described [1,16,17,18,19,20]. In the family herein presented, the rearrangement segregates through three generations, with six individuals harboring the balanced rearrangement and three individuals harboring the derivative chromosome 9. Consequently, the latter present a large chromosome 15 duplication of 19.3 Mb at q21.1q22.31 region. Considering that independent synapsing of homologous pair occurs, two unbalanced combinations would be expected, one with duplication and the other with deletion of the insertional segment. Interestingly, no individuals harboring the deletion of 15q21.1q22.31 were found in this family. Similarly, no patient with a deletion with these breakpoints is described in the DECIPHER database. Possibly, the conceptuses harboring this deletion may not be viable. Of note, only one female carrier of the balanced insertion in this family (individual III-9) had two consecutive abortions. 

**Figure 4:**
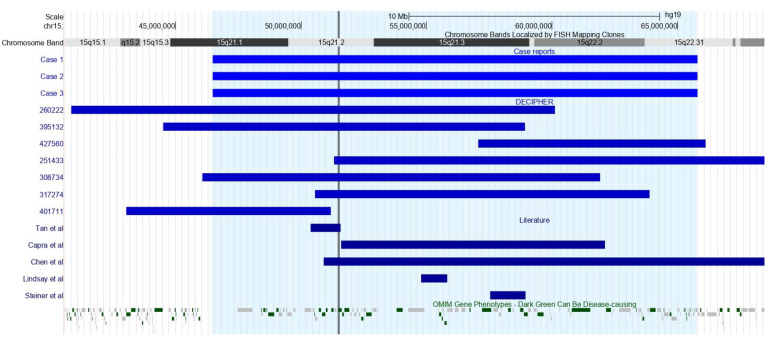
View of the 15q15.1-q26.3 region, including duplications, represented by blue bars, from the present subjects and cases from DECIPHER and literature. The *CYP19A1* gene region is highlighted by the black vertical line. Image taken from UCSC Genome Browser on Human (GRCh37/hg19).

To date, few cases of chromosomal insertions involving chromosome 15 have been described [1,21,22,23,24]. Chromosome 15 is one of the seven human chromosomes with the highest rates of segmental duplications, which makes it more susceptible to chromosomal rearrangements [25]. However, we found few cases in the literature presenting duplications that overlap with the 15q21.1-q22.31 region. Only five cases published were found in the literature [5,6,7,8,9], and seven cases in DECIPHER (260222; 395132; 427560; 251433; 308734; 317274; and 401711) (Figure 4). The most frequent clinical features found among patients harboring duplications between 15q15.1 and q26.3 regions were short stature (9/15); intellectual disability (8/15); delayed speech and language development (8/15); neurodevelopmental delay (7/15); congenital heart disease (8/15); low-set ears (7/15); and facial dysmorphisms (6/15) (Table 1).

The duplicated region of the patients presented in this study contains 157 genes, among which 95 are registered in the OMIM Database. Tan and coworkers (2019) [7] reported a patient with a 1.1 Mb duplication at 15q21.2 region (50,382,769–51,568,204)(hg19) involving the *CYP19A1* gene (OMIM # 107910), mapped within the 15q21.2 region (51,208,057–51,338,596)(hg19), which is associated with the Aromatase Excess Syndrome (OMIM # 139300) when expression of *CYP19A1* is increased. Due to excessive estrogen formation, clinical manifestations in boys include prepubertal gynecomastia, decreased testicular size, accelerated skeletal maturation, and premature fusion of epiphyses, resulting in short stature in adulthood [26,27]. In girls, the manifestations include premature thelarche, breast hypertrophy, enlarged uterus, and irregular menstruation [28]. All subjects harboring 15q21.2 duplication in this study presented with short stature (subjects 1, 2, and 4). Regarding male patients of the literature, gynecomastia was reported in only one individual [7], whereas decreased testicular size and micropenis were identified in DECIPHER cases 395132 and 260222, respectively. Subject 4 herein described presented with accelerated skeletal maturation, but he did not allow genital examination. Despite this, no endocrinological abnormalities were detected. Among female patients, subjects 1 and 2 of the present study and DECIPHER case 308734 showed abnormality of the menstrual cycle, secondary amenorrhea, premature ovarian insufficiency, and early onset of sexual maturation. Thus, the increased expression of the *CYP19A1* gene could be responsible for short stature and irregular menstruation in female patients.

Among the individuals with the balanced rearrangement in the family herein presented, no phenotypic alterations were found, except for subject 3, a female patient who inherited the rearrangement from her mother. She had microcephaly, severe intellectual disability, absent speech, and ataxia. Since genomic imbalances can be found in up to 30–50% of individuals with apparently balanced chromosomal rearrangements and abnormal phenotype, we performed CMA, which did not reveal pathogenic imbalances in this patient. Following diagnostic investigation, low-pass WGS was performed searching for disrupted genes at the breakpoints, which could be associated with the phenotype. No genes were found at the breakpoints on chromosome 15, and the breakpoint at 9q33 disrupted the *RABGAP1* gene. Recently, biallelic loss-of-function variants in this gene were found to cause a novel neurodevelopmental syndrome [29]. Even though the disruption of this gene causes its loss of function, the mode of inheritance is not compatible, since only one allele is affected in the patient presented here. In addition, a study published by Aristidou et al. (2018) [30], performing WGS for accurate breakpoint mapping in apparently balanced translocation families with discordant phenotypes, showed that identical breakpoints were identified in affected and non-affected members carrying the same translocations [30]. Therefore, it should be expected that the disruption of the *RABGAP1* gene occurs in all family members harboring the balanced insertion, and most of them have normal phenotypes. 

Further diagnostic investigation of subject 3, using WES, identified a pathogenic small deletion in the *MECP2* gene (OMIM#300005). Although Rett syndrome is a well-known genetic disorder, at the beginning of the diagnostic investigation, the clinical features of this patient did not suggest this hypothesis, because some features started more recently. In addition, the phenotype of this patient was compatible with atypical Rett Syndrome. 

## 5. Conclusions

This study contributes to the description of the clinical features associated with the 15q21.1q22.31 duplication. Moreover, it reinforces that the diagnostic investigation of individuals with abnormal phenotypes—carriers of inherited balanced chromosomal rearrangements with discordant phenotypes—should include genomic tests, such as WES, and should not be focused on the chromosome breakpoints because, in these cases, other genetic events may be responsible for the phenotype instead of the chromosomal rearrangement.

## Figures and Tables

**Table 1:** Main clinical features of patients with duplication between the 15q15.1-q26.3 regions.

	Present Case Reports			Cases From Literature					Cases From DECIPHER							
	Subject 1	Subject 2	Subject 4	[7]	[5]	[9]	[6]	[8]	260222	395132	427560	251433	308734	317274	401711	Total
Gender	female	female	male	male	famale	male	male	male	male	male	male	female	female	male	female	
Chromosome region	15q21.1q22.31	15q21.1q22.31	15q21.1q22.31	15q21.2	15q21.2q22.2	15q21.2q26.3	15q21.3	15q21.3	15q15.1q22.2	15p21.1q22.1	15q21.3q22.31	15q21.2q24.1	15q21.1q22.2	15q21.2q22.31	15q15.2q21.2	
Genomic positions	chr15:46,487,891–65,795,296	chr15:46,487,891–65,795,296	chr15:46,487,891–65,795,296	chr15:50,382,769–51,568,204	chr15:51,604,508–62,102,756	chr15:50,903,432–102,338,129	chr15:57,529,846–58,949,448	chr15:54,776,491–55,822,045	chr15:40,848,086–60,105,306	chr15:44,520,510–58,920,509	chr15:57,052,168–66,127,560	chr15:51,314,381–73,572,490	chr15:46,072,402–61,905,184	Chr15:50,554,375–63,890,439	chr15:43,032,935–51,179,063	
Duplication size (Mb)	19.3	19.3	19.3	1.1	10.5	2.4	1.4	1.0	19.3	14.4	9.1	22.3	15.8	13.3	8.2	
Intellectual disability	+	+	+	NR	−	NR	NR	−	+	NR	+	−	+	+	+	08/15
Neurodevelopmental delay	+	+	+	NR	+	NR	NR	+	NR	NR	+	−	−	+	−	07/15
Delayed speech and language development	+	+	+	NR	−	NR	NR	+	+	NR	+	+	−	+	−	08/15
Abnormal heart morphology	+	+	+	NR	−	NR	+	+	+	NR	NR	+	+	NR	NR	08/15
Short stature	+	+	+	+	NR	NR	NR	−	+	+	−	+	+	NR	+	09/15
Cubitus valgus	+	+	+	+	NR	NR	−	−	NR	NR	NR	NR	NR	NR	NR	04/15
Abnormal facial shape	+	+	+	NR	−	+	−	−	NR	+	−	−	NR	+	+	07/15
Strabismus	−	−	+	NR	−	NR	−	+	NR	+	−	−	NR	−	−	03/15
Low-set ears	+	+	+	NR	−	+	−	−	NR	+	+	+	NR	−	−	07/15
Accelerated skeletal maturation	+	+	+	+	NR	NR	NR	NR	NR	NR	NR	NR	NR	NR	NR	04/15
Other features	Premature ovarian insufficiency.	Secondary amenorrhea.	Abnormal skeletal muscle morphology; Skeletal muscle hypertrophy; Velopharyngeal insufficiency; Hypothyroidism.	Gynecomastia.	Vascular malformation in the left maxillary sinus;		Fetal omphalocele; Tetralogy of Fallot.	Anxiety; Obsessive compulsive tendencies; Inability to walk; Motor stereotypy.	Micropenis; Delayed cranial suture closure; Seizures; Delayed skeletal maturation.	Cryptorchidism; Hypospadias; Small scrotum; Obesity.	Hypotonia.	Ptosis.	Early onset of sexual maturation.		Macrotia; Hypertelorism; Proptosis; Pes planus; Delayed skeletal maturation.	

NR = not reported; + = present; − = absent.

## Data Availability

The data that support the findings of this study are available from the corresponding author upon reasonable request.
